# Differentiating Fat-Poor Angiomyolipoma from Renal Cell Carcinoma Using Contrast-Enhanced CT

**DOI:** 10.3390/bioengineering13090967

**Published:** 2026-08-24

**Authors:** Jinglai Lin, Letong Zhang, Dengqiang Lin, Linpeng Yao, Kang Wang, Ying Xiong

**Affiliations:** 1 Department of Urology, Zhongshan Hospital (Xiamen), Fudan University, Xiamen 361015, China; 2 Xiamen Clinical Research Center for Cancer Therapy, Zhongshan Hospital (Xiamen), Fudan University, Xiamen 361015, China; 3 Clinical Research Center for Precision Medicine of Abdominal Tumor of Fujian Province, Zhongshan Hospital (Xiamen), Fudan University, Xiamen 361015, China; 4 Department of Urology, Urology Research Institute, The First Affiliated Hospital, Fujian Medical University, Fuzhou 350009, China; 5 Clinical Medical College, Chengdu University, Chengdu 610086, China; 6 Department of Radiology, The First Affiliated Hospital, Zhejiang University School of Medicine, Hangzhou 310000, China; 7 Digital Medical Research Center, School of Basic Medical Sciences, Fudan University, Shanghai 200000, China; 8 Department of Urology, Zhongshan Hospital, Fudan University, Shanghai 200000, China

**Keywords:** fat-poor angiomyolipoma, renal cell carcinoma, radiomics, contrast-enhanced computed tomography, diagnosis

## Abstract

Fat-poor angiomyolipoma (fp-AML), a common benign renal mass, closely mimics renal cell carcinoma (RCC) on preoperative computed tomography (CT), frequently resulting in unnecessary surgical intervention. This multicenter retrospective study aimed to develop and externally validate an AI-assisted radiomics model based on triphasic contrast-enhanced CT to accurately distinguish fp-AML from RCC. A total of 655 eligible patients with sporadic solid renal lesions were enrolled and divided into a training cohort (*n* = 364), an internal test cohort (*n* = 156), and an independent external validation cohort (*n* = 135), with a stable fp-AML to RCC ratio of approximately 1:4. Tumor segmentation was performed using an nnU-Net-assisted workflow with radiologist refinement, followed by cross-phase image registration, radiomic feature extraction, LASSO feature reduction, and random forest classifier construction. The established model achieved favorable and stable diagnostic performance across all cohorts, with AUCs of 0.868 in the training and internal test sets and 0.803 in the external validation set, maintaining reliable discrimination even in the small renal mass subgroup (≤4 cm). This externally validated AI radiomics model demonstrates promising diagnostic performance across centers and may serve as a preoperative decision-support tool for differentiating fp-AML from RCC; however, prospective multicenter validation is required before routine clinical implementation.

## 1. Introduction

With the increasing use of cross-sectional imaging, an increasing number of renal masses are detected incidentally during examinations performed for unrelated medical reasons [1,2]. However, earlier detection of renal masses has not translated into improved cancer-specific survival, suggesting potential overtreatment [3]. Among renal lesions resected surgically, approximately 20% are benign, and fat-poor angiomyolipoma (fp-AML) is one of the most common benign subtypes; nevertheless, fp-AML is frequently treated unnecessarily with partial or radical nephrectomy [4]. Most angiomyolipomas (AMLs) containing macroscopic intratumoral fat can be readily identified on computed tomography (CT) or magnetic resonance imaging (MRI). By contrast, fp-AML accounts for approximately 5% of all AMLs, and its minimal fat content makes it difficult to distinguish from renal cell carcinoma (RCC) on CT or MRI [5,6]. Accurate differentiation between fp-AML and malignant RCC is therefore essential to avoid unnecessary surgery. Unfortunately, establishing a reliable preoperative diagnosis of fp-AML remains challenging for both radiologists and urologists. CT is the most commonly used imaging modality for evaluating renal masses. Previous studies have proposed conventional CT features that may help differentiate fp-AML from RCC, including hyperattenuation on non-contrast CT, homogeneous delayed enhancement, uniform texture, and wedge-shaped or round morphology [7,8,9]. However, these differences are often subtle on visual assessment, and considerable feature overlap exists between fp-AML and malignant RCC [10,11].

Radiomics converts medical images into high-dimensional, mineable data and has been applied to the management of renal tumors, including prediction of nuclear grade, discrimination of RCC subtypes, and prognostic assessment [12,13,14]. Newer image-analysis approaches, including radiomics and artificial intelligence (AI), have also shown promise for distinguishing fp-AML from RCC on contrast-enhanced imaging [15,16,17,18]. Texture analysis has been explored as a preoperative tool; for example, Hodgdon and colleagues supported the use of quantitative texture analysis on unenhanced CT for predicting fp-AML [19]. However, many prior studies lacked validation in independent cohorts, which may substantially overestimate performance. In addition, earlier work often included only selected RCC histologic subtypes—predominantly clear cell RCC (ccRCC)—whereas non-ccRCC subtypes may also be confused with fp-AML. Here, we aimed to develop a learning-based model to differentiate fp-AML from RCC and to validate it in an independent cohort. We included size-matched RCC cases across all histologic subtypes to better reflect real-world diagnostic difficulty.

## 2. Materials and Methods

### 2.1. Patient Selection

This multicenter retrospective study was conducted in accordance with the Declaration of Helsinki and approved by the ethics committees of Zhongshan Hospital, The First Affiliated Hospital of Zhejiang University, Lianyungang People’s Hospital, Xiamen Hospital, and Lianyungang First People’s Hospital. The requirement for informed consent was waived. We retrospectively enrolled patients who underwent partial or radical nephrectomy for renal masses at the participating institutions: Zhongshan Hospital (January 2009 to March 2022), The First Affiliated Hospital of Zhejiang University (January 2012 to December 2017), Lianyungang People’s Hospital (April 2017 to June 2018), and Xiamen Hospital and Lianyungang First People’s Hospital (September 2017 to March 2022). Inclusion criteria were (1) nonmetastatic, sporadic, solid renal lesions treated surgically; (2) preoperative triphasic CT including non-contrast (N-phase), arterial (A-phase), and venous (V-phase) scans; and (3) postoperative pathology confirming RCC of any histologic subtype or fp-AML without visible macroscopic fat on imaging. Exclusion criteria were (1) severe artifacts or marked noise preventing reliable tumor segmentation; (2) incorrect scan delay timing after contrast injection on CT; (3) bilateral or multifocal renal lesions, or metastatic RCC; and (4) tumor rupture with hemorrhage, perirenal fat stranding, and/or perirenal fluid collection. Histologic subtypes were re-evaluated according to the 2016 WHO Classification of Tumours of the Urinary System and Male Genital Organs [20], and tumor stage was assigned according to the eighth edition of the AJCC TNM staging system (2017). To address class imbalance and ensure an adequate number of fp-AML cases for model development, a prespecified fp-AML-to-RCC sampling ratio of approximately 1:4 was adopted. This ratio was used as a class-sampling strategy and was not intended to reproduce the actual prevalence of fp-AML in routine clinical practice. The training and internal test cohorts were subsequently generated using stratified random sampling to preserve the class proportions, while an independent external cohort was used for validation (training set, n = 364; internal test set, n = 156; external test set, n = 135).

### 2.2. CT Acquisition and Image Preprocessing

All included cases had preoperative triphasic CT (non-contrast, arterial, and venous phases). All CT images were retrieved from the Picture Archiving and Communication System (PACS) of the five participating hospitals. The acquisition parameters are summarized in Table S1. Contrast protocol: The general CT procedure consisted of first performing a routine unenhanced scan. This was followed by intravenous injection of a contrast agent (1.5–2 mL/kg) into an antecubital vein at an injection rate of 2.5–4 mL/s using a power injector. Two postcontrast phases were obtained: the corticomedullary phase at 25–40 s after the start of injection and the nephrographic phase at 50–95 s after injection.

Prior to analysis, all DICOM images were converted to the NIfTI format for de-identification, thereby removing all patient-identifying metadata before image processing. For the segmentation stage, the arterial-phase CT images were preprocessed using the standard preprocessing pipeline adopted by nnU-Net for the KiTS dataset [21]. The resulting tumor segmentation masks were used as the regions of interest (ROIs). Using these ROIs, the multi-phase CT images were cropped and rigidly registered to the arterial-phase images using linear interpolation. Subsequently, the axial slice containing the largest tumor area was extracted from each registered imaging volume, and the in-plane voxel spacing was resampled to 0.625 × 0.625 mm using linear interpolation. For intensity normalization, image intensities were clipped to the range of −500 to 500 Hounsfield units (HU) and then normalized to the range of 0–1 before feature extraction. During radiomic feature extraction, a bin width of 4 was used for gray-level discretization. No image filtering was applied before feature extraction, and radiomic features were extracted only from the original CT images.

### 2.3. Tumor Segmentation

To capture intratumoral heterogeneity, tumors were delineated slice by slice on arterial-phase CT images to generate ROIs for subsequent radiomics analysis and model development. Specifically, experienced radiologists manually delineated the kidneys and lesion regions on a subset, which contained 100 cases of arterial-phase CT images from the entire dataset. These annotations were used to train an nnU-Net-based automatic segmentation model capable of identifying renal tumor regions on arterial-phase CT images [21]. Specifically, we employed a 3D nnU-Net initialized with the pretrained weights released for the KiTS dataset [22] and fine-tuned the model for 100 epochs using our local dataset of 100 arterial-phase CT scans with pixel-wise ground-truth annotations. This training strategy was consistent with the standard segmentation pipeline adopted in our previous study. As reported previously [23], the resulting segmentation model was evaluated on an independent cohort of 50 arterial-phase CT scans and achieved Dice similarity coefficients of 0.968 and 0.852 for kidney and renal tumor segmentation, respectively, demonstrating good agreement with expert annotations. The trained nnU-Net model was then applied to automatically segment regions containing renal masses, and the resulting segmentations were manually refined by a senior radiologist to obtain high-quality ROI annotations. Thereafter, the General Registration tool in 3D Slicer [24,25] was used to register and align CT images from the other two phases (venous and non-contrast) to the arterial-phase images, enabling extraction of corresponding ROI regions across all three phases.

### 2.4. Model Construction

#### 2.4.1. Radiomics Feature Extraction

Radiomics features were extracted from CT images within 2D ROIs across all three imaging phases, including first-order statistics and multiple texture feature families: gray-level co-occurrence matrix (GLCM), gray-level dependence matrix (GLDM), gray-level run-length matrix (GLRLM), gray-level size-zone matrix (GLSZM), and neighborhood gray-tone difference matrix (NGTDM), thereby comprehensively characterizing intensity distribution and texture heterogeneity. Specifically, radiomics features were extracted from the three imaging phases using the PyRadiomics Python package (version 3.1.0) [26] on the basis of the corresponding ROI annotations. Features were extracted from the axial slice showing the maximum tumor diameter. For each imaging phase, the extracted features included shape features (nine two-dimensional geometric features describing the maximum tumor dimension on the axial slice), first-order histogram-based statistical features, and texture features derived from the GLCM, GLRLM, GLSZM, GLDM, and NGTDM. In total, 100 radiomics features were extracted for each CT phase (non-contrast, arterial, and venous). After removal of redundant shape features shared across phases, 282 radiomics features were retained for subsequent feature selection and analysis.

#### 2.4.2. Feature Selection and Classifier Training

We formulated differentiation of fp-AML and RCC as a supervised binary classification task, with the pathological diagnosis of fp-AML or RCC used as the class label. The same radiomics feature-extraction procedure was applied to both fp-AML and RCC lesions. Feature selection and classifier construction were implemented using the scikit-learn Python package [27].

Among the 282 candidate radiomics features extracted from the three CT phases, LASSO regression was performed in the training cohort to identify features associated with the pathological class labels while removing irrelevant or redundant features. The LASSO hyperparameter λ (α) was set to 0.001. Features with coefficients equal to zero were excluded, and the remaining features were ranked according to their coefficients. Ultimately, 25 features were selected as inputs to the predictive model.

Before feature selection, all extracted features were standardized using z-score normalization to ensure comparable distributions across the dataset. Given its strong nonlinear classification capability, robustness in handling high-dimensional radiomic features, resistance to overfitting, and a previous study [23] that demonstrated that the RF classifier achieved reliable and competitive performance for CT-based renal tumor classification, a Random Forest (RF) classifier was selected as the final prediction model. The RF classifier was implemented using the scikit-learn Python package. Specifically, the number of decision trees was set to 100, the random seed was fixed at 1 to ensure reproducibility, and all other hyperparameters were kept at their default values. The classifier was trained to discriminate fp-AML from RCC and generated a probability score between 0 and 1, which was subsequently converted into a binary prediction.

#### 2.4.3. Threshold Determination and Performance Evaluation

For the RF model, training was performed on the training dataset, followed by selection of the optimal classification probability threshold on the internal test set. The optimal cutoff was determined by maximizing the Youden index in the internal test set. Model performance was primarily evaluated using receiver operating characteristic (ROC) curves and the area under the ROC curve (AUC). The 95% confidence intervals (CIs) for the AUC were obtained using 1000 bootstrap resampling iterations. Accuracy (ACC), sensitivity (recall), specificity, and F1 score were also reported for the training, internal test, and external test sets as complementary performance metrics. Decision curve analysis (DCA) was performed to evaluate the potential clinical utility of the radiomics model by calculating the net benefit across a range of threshold probabilities. RCC was defined as the positive outcome, and the model was compared with the default strategies of treating all patients as having RCC and treating none as having RCC. DCA was performed in the external test cohort and additionally in the small renal mass subgroup (≤4 cm).

To improve model interpretability, SHAP (SHapley Additive exPlanations) analysis was performed using the SHAP Python package. Global feature importance was quantified using the mean absolute SHAP value, while SHAP summary (beeswarm) plots were generated to visualize the direction and magnitude of each feature’s contribution across all samples.

### 2.5. Statistical Analysis

Statistical analyses were performed using SPSS Statistics 22.0 and R version 4.1.1. ROC analysis was used to evaluate diagnostic performance. Student’s t-test was used to compare predicted fp-AML scores across subgroups. All tests were two-sided, and *p* < 0.05 was considered statistically significant.

## 3. Results

### 3.1. Flowchart of Patient Recruitment and Clinicopathologic Characteristics

**Figure 1:**
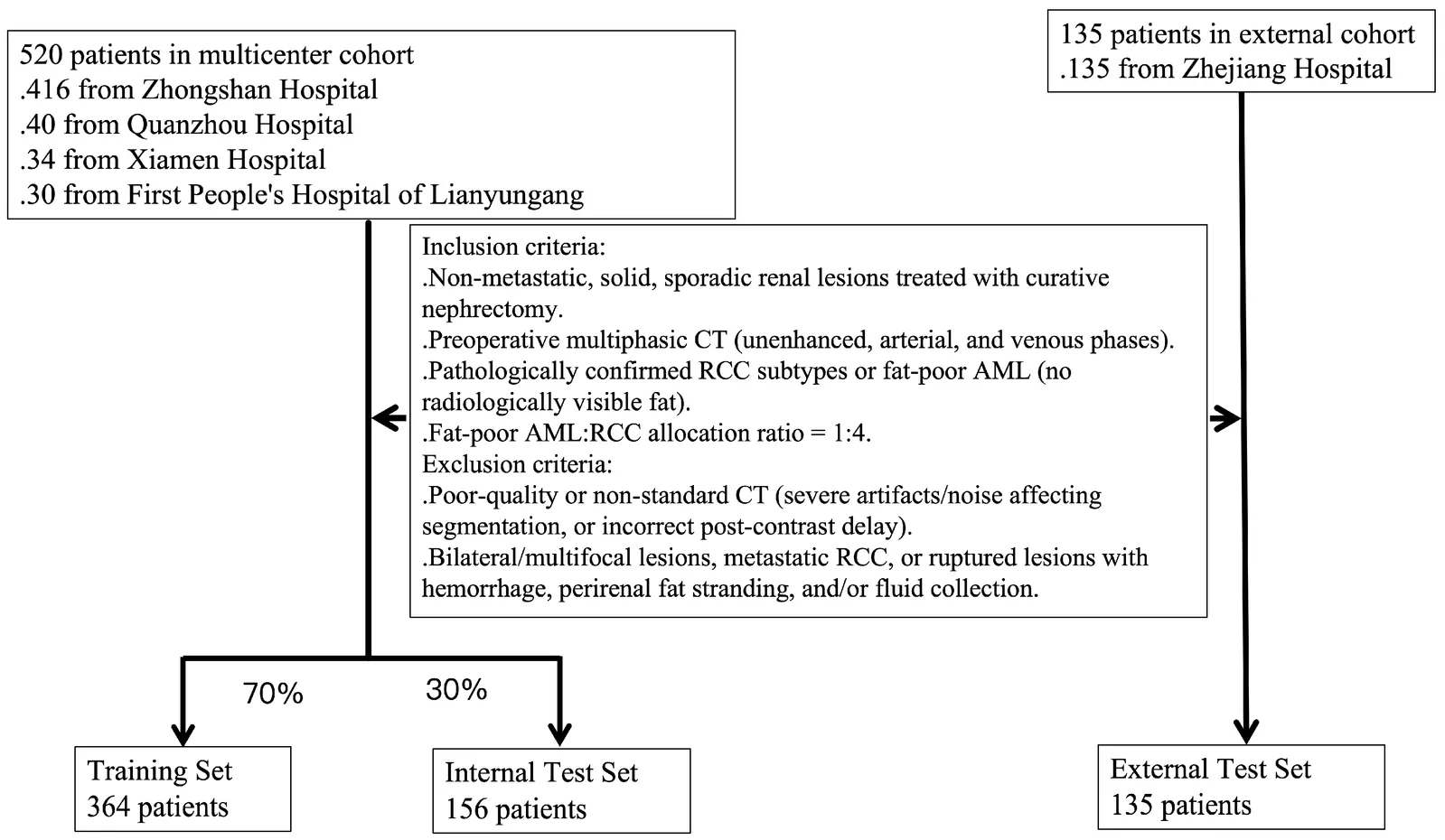
Patient recruitment.

**Figure 2:**
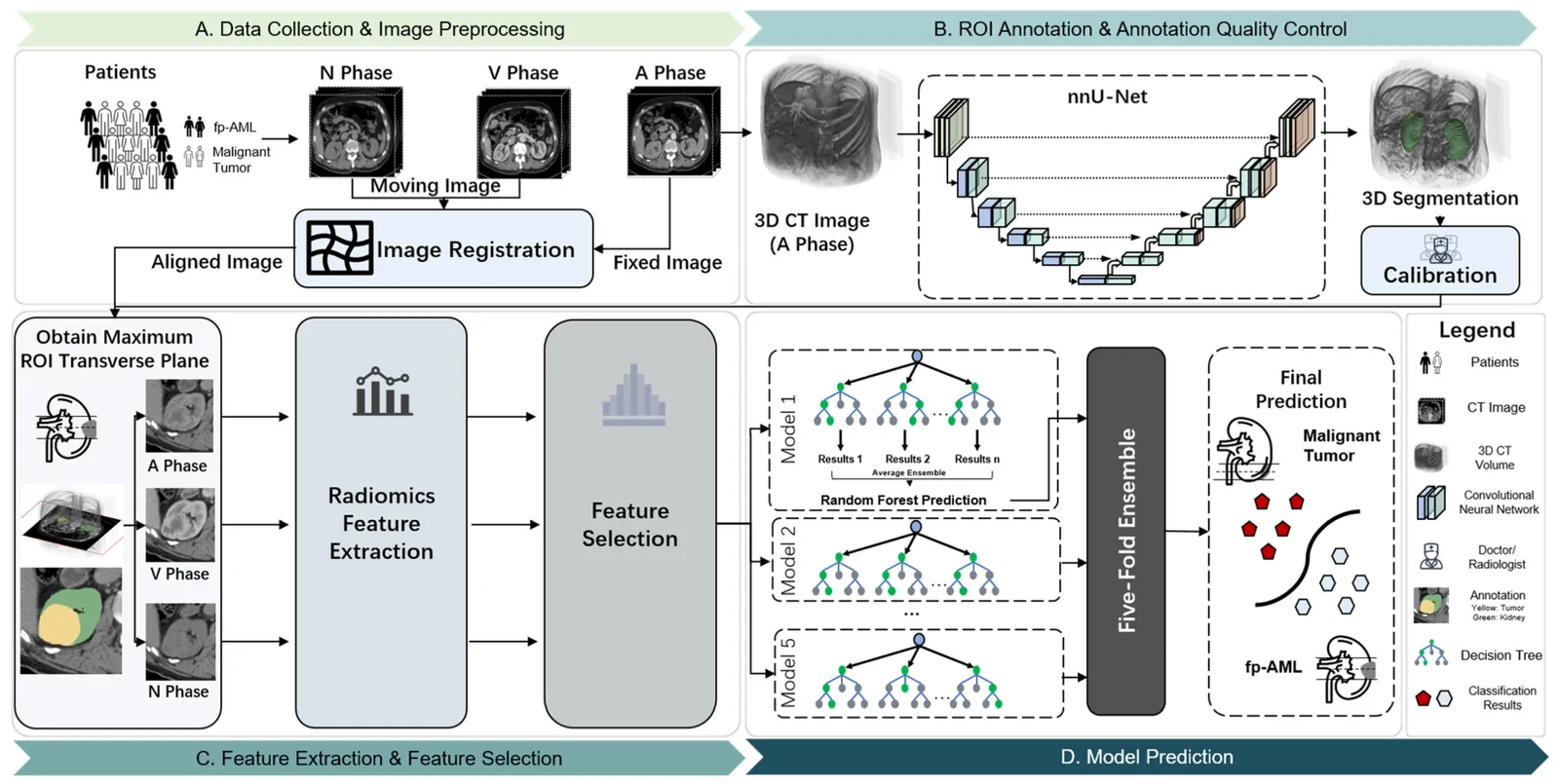
**Overall workflow of the study.** A. Data collection & image preprocessing. B. ROI annotation and calibration. C. Feature extraction and feature selection. D. Model prediction.

The overall workflow of the present study and detailed patient recruitment procedures were systematically illustrated in Figure 1 and Figure 2, respectively. Briefly, the analytical pipeline was sequentially implemented following four core procedures, including raw clinical data acquisition and computed tomography (CT) image standardized preprocessing, region of interest (ROI) manual annotation and quantitative calibration, high-dimensional radiomic feature extraction and dimensionality reduction-based feature screening, and final predictive model construction and validation. The final dataset included 364 cases in the training set, 156 in the internal test set, and 135 in the external test set, corresponding to 1092, 468, and 405 triphasic CT volumes, respectively. Zhongshan Hospital (n = 416), Lianyungang People’s Hospital (n = 40), Xiamen Hospital (n = 34), and Lianyungang First People’s Hospital (n = 30) contributed cases to the training and internal test sets, whereas all 135 cases from The First Affiliated Hospital of Zhejiang University comprised the external test set. Median age (IQR) was 58 (50–67), 55.5 (48–64), and 56 (48–63) years across the three cohorts, and the proportion of male patients was 56.6%, 62.2%, and 63.7%, respectively. Partial nephrectomy was the predominant surgical approach (62.9%, 62.2%, and 68.9%), whereas radical nephrectomy accounted for approximately one-third of cases (35.7%, 35.9%, and 29.6%); a small fraction underwent needle biopsy (1.4%, 1.9%, and 1.5%). Median tumor diameter (IQR) was 3.0 (2.0–4.7) cm, 3.5 (2.2–5.0) cm, and 3.2 (2.1–4.3) cm in the training, internal test, and external test sets, respectively; small renal masses (≤4 cm) predominated (70.9%, 63.5%, and 74.1%). Nearly all lesions were solid on imaging (99.5%, 99.4%, and 100%, respectively). Most tumors were TNM stage I (84.5%, 81.6%, and 90.8%), with no stage IV cases. Clear cell RCC was the most common subtype (approximately 69–72%), and papillary RCC, chromophobe RCC, and other rare subtypes were also included. fp-AML accounted for 20.1%, 19.9%, and 19.3% of the three cohorts, consistent with the prespecified sampling ratio (Table 1).

### 3.2. Radiomics Feature Selection

**Figure 3:**
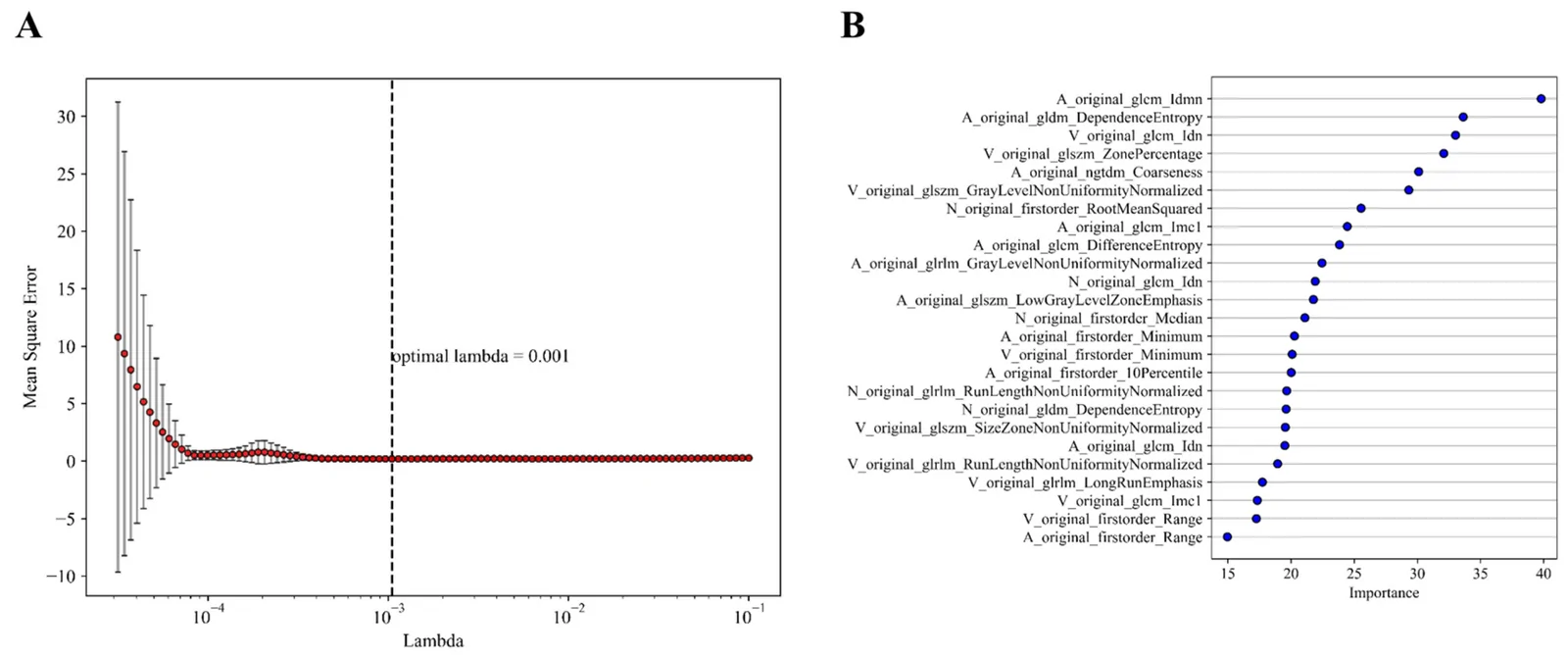
**Feature selection by Lasso regression analysis.** (**A**) Selection of the optimal lambda value. (**B**) Top 25 radiomics features selected by LASSO regression.

Radiomics features were extracted from 2D ROIs on non-contrast (N), arterial (A), and venous (V) phase CT and reduced using LASSO regression (λ = 0.001). Twenty-five features with non-zero coefficients were retained for classification modeling (Figure 3; Table S2). The selected features spanned first-order statistics and multiple texture families (GLCM, GLDM, GLRLM, GLSZM, and NGTDM), indicating that intensity distribution and multiscale texture heterogeneity contain informative signals for differentiating fp-AML from RCC (Supplementary Table S2). The selected features also captured enhancement-related information across phases, including arterial- and venous-phase texture-structure metrics, region/dependence complexity indices, and non-contrast intensity statistics, suggesting that complementary multiphasic information jointly contributes to differential diagnosis (Table S2).

### 3.3. Diagnostic Performance and External Validation

**Figure 4:**
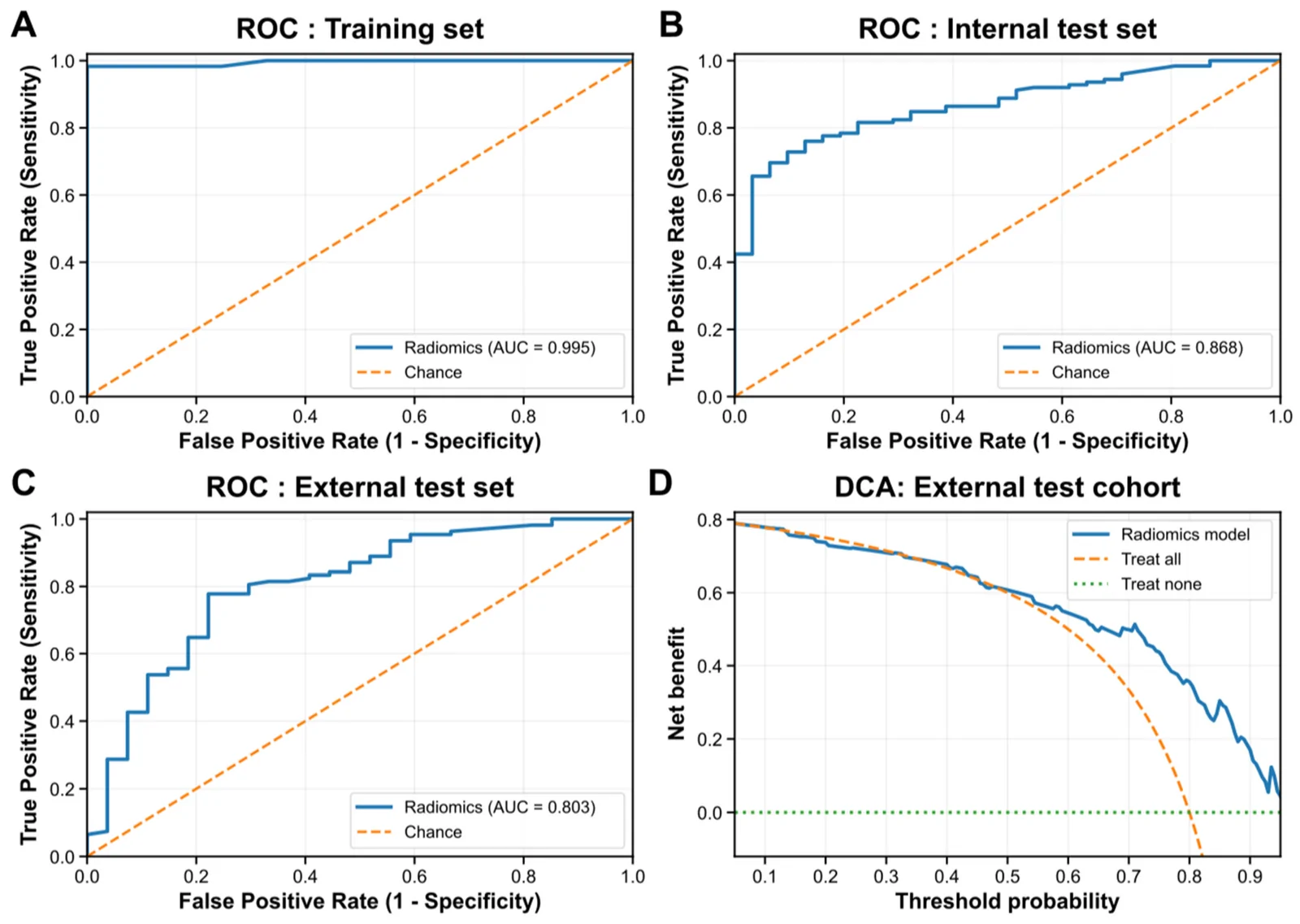
**Diagnostic performance of the model across different cohorts.** (**A**) ROC curves in training set. (**B**) ROC curves in internal test set. (**C**) ROC curves in external test set. (**D**) Decision curve analysis of the radiomics model in the external test cohort compared with the treat-all and treat-none strategies.

A random forest classifier was built using the 25 selected radiomics features. ROC curves for the training, internal test, and external test sets are shown in Figure 4A–C. To convert probability outputs into binary predictions, the optimal threshold was determined in the internal test set by maximizing the Youden index (optimal threshold = 0.73) (Table 2). In the training set, the model achieved an AUC of 0.868 (95% CI: 0.748–0.940), ACC of 91.4%, sensitivity of 70.6%, specificity of 100%, and F1 score of 0.87 (Table 2). In the internal test set, discrimination remained high, with an AUC of 0.868 (95% CI: 0.712–0.948), ACC of 74.4%, sensitivity of 69.6%, specificity of 93.5%, and F1 score of 0.813 (Table 2). In the independent external test set, the model demonstrated stable generalizability, with an AUC of 0.803 (95% CI: 0.741–0.865), ACC of 73.3%, sensitivity of 72.2%, specificity of 100%, and F1 score of 0.90 (Table 2). Overall, the radiomics model maintained robust cross-center performance, particularly with high specificity (Figure 4, Table 2). Decision curve analysis demonstrated that the radiomics model provided a greater net benefit than the treat-all and treat-none strategies across a broad range of threshold probabilities in the external test cohort (Figure 4D). At the selected probability threshold of 0.73, the net benefit of the model was 0.458, compared with 0.259 for the treat-all strategy.

### 3.4. Performance in Small Renal Masses

Because small renal masses predominated (Table 1), we further evaluated model performance in this subgroup. ROC curves are shown in Figure S1A–C. The model achieved an AUC of 0.996 (95% CI: 0.991–1.000) in the training set and maintained stable discrimination in the internal and external test sets (internal: AUC, 0.808, 95% CI: 0.718–0.889; external: AUC, 0.799, 95% CI: 0.667–0.915). Although performance decreased relative to the training set, the similar AUCs across the two independent test cohorts suggest potential utility in the most common clinical scenario. DCA in the small renal mass subgroup similarly showed that the radiomics model provided a greater net benefit than the treat-all and treat-none strategies across a broad range of threshold probabilities in the external test cohort (Figure S1D). At the selected threshold of 0.73, the net benefit of the model was 0.459, compared with 0.296 for the treat-all strategy.

### 3.5. Model Interpretability Based on SHAP Analysis

SHAP analysis was performed to improve the interpretability of the proposed random forest model. As shown in Figure S2A, arterial-phase GLDM Dependence Entropy, GLSZM Low Gray Level Zone Emphasis, and GLRLM Gray Level Non-Uniformity Normalized were the three most influential features according to the mean absolute SHAP value, indicating that arterial-phase texture descriptors contributed most substantially to model predictions. Features derived from the non-contrast and venous phases generally exhibited lower global importance. The SHAP summary plot (Figure S2B) further illustrated the direction and magnitude of feature contributions across individual patients. Higher values of GLDM Dependence Entropy and non-contrast GLCM IDN indicated an increased predicted probability of the positive class (malignant renal tumor), whereas higher values of GLSZM Low Gray Level Zone Emphasis, GLRLM Gray Level Non-Uniformity Normalized, and NGTDM Coarseness indicated a decreased predicted probability of the positive class, equivalently, and shifted predictions toward fp-AML. The clear separation of high- and low-value samples among the top-ranked features indicates consistent contributions across the study population, whereas lower-ranked features exhibited weaker and more heterogeneous effects.

### 3.6. Calibration Analysis

We further evaluated the calibration performance of the proposed model in the independent external cohort. Calibration curves (Figure S3) were generated by comparing predicted probabilities from the original random forest model with the observed frequencies of malignant tumors. No additional recalibration was performed to avoid introducing information leakage from the external test cohort. Calibration analysis showed reasonable agreement between predicted probabilities and observed outcomes in the external test cohort (Brier score = 0.134). The calibration intercept was 0.686 and the calibration slope was 0.729, indicating a slight underestimation of absolute risk and moderate overconfidence of predicted probabilities.

## 4. Discussion

In this study, we developed and validated an AI-assisted differential diagnostic model based on preoperative triphasic contrast-enhanced CT to distinguish fp-AML from RCC. In the independent external validation cohort, the model achieved a sensitivity of 72.2%, specificity of 100%, and diagnostic accuracy of 73.3%. The robust performance and notably high specificity across different centers suggest that this radiomics-based tool can provide reliable, standardized quantitative support for preoperative decision-making. Furthermore, DCA supported the potential clinical utility of the model by demonstrating a higher net benefit than the treat-all and treat-none strategies in the external test cohort, with a similar pattern observed among small renal masses.

SHAP analysis revealed that arterial-phase texture descriptors contributed most substantially to model predictions, with GLDM Dependence Entropy ranked as the most influential feature. Higher values of this feature indicated an increased predicted probability of RCC, whereas lower values indicated a decreased predicted probability of RCC, equivalently, and shifted predictions toward fp-AML. Dependence Entropy measures the complexity and heterogeneity of voxel dependency patterns within the lesion. The contribution of higher Dependence Entropy to RCC prediction may reflect the greater intratumoral heterogeneity associated with necrosis, hemorrhage, fibrosis, and irregular vascular architecture. Conversely, the lower entropy and more uniform arterial-phase texture associated with fp-AML may reflect its relatively homogeneous internal architecture and enhancement pattern. By accurately identifying benign fp-AML, the model may help clinicians avoid unnecessary radical or partial nephrectomy, particularly for incidentally detected small renal masses.

Radiomics-enhanced interpretation of contrast-enhanced CT has been proposed as a promising approach for distinguishing fp-AML from RCC [28,29]. Cui and colleagues developed a machine-learning model using 41 fp-AML and 130 RCC cases and reported an AUC of 0.96, outperforming radiology experts [18]. Yang and colleagues evaluated 224 classifiers using 118 RCC and 45 fp-AML cases; the best model based on non-contrast CT achieved an AUC of 0.90 [17]. In a study of 39 fp-AML and 41 clear cell RCC (ccRCC) cases, a machine-learning classifier outperformed a radiomics model, with sensitivity of 77% and specificity of 76% [30,31]. However, these models generally lacked external validation. Moreover, many studies included only major RCC subtypes (ccRCC, papillary RCC, and chromophobe RCC), which may introduce selection bias and do not fully reflect real-world diagnostic difficulty, because non-ccRCC subtypes may also be confused with fp-AML. In addition, most prior work analyzed only one to four representative slices rather than the entire tumor extent, which may inadequately capture intratumoral heterogeneity. Compared with previous studies, our work included a larger cohort and was validated in an independent medical center, supporting reproducibility and generalizability. By reclassifying tumors according to the 2016 WHO criteria and including a broader range of RCC histologies, we partially mitigated selection bias.

Several limitations should be noted. First, the model was developed using surgically resected, pathologically confirmed cases, which may introduce selection bias because surgically treated tumors are often considered more suspicious for malignancy or invasiveness. Although multicenter inclusion may reduce single-center decision bias, prospective validation in broader real-world populations is warranted. Second, the prespecified fp-AML-to-RCC ratio of 1:4 resulted in an enriched proportion of fp-AML cases compared with routine clinical populations. This sampling strategy was adopted to ensure an adequate number of minority-class cases for model development and evaluation rather than to reproduce the actual disease prevalence. Consequently, although the model demonstrated favorable discrimination in the external test cohort, its predicted probabilities, calibration, predictive values, optimal threshold, and clinical net benefit may differ when applied to populations with a lower prevalence of fp-AML. In addition, radiomic feature reproducibility was not evaluated using ICC or test–retest analyses. Given the multicenter nature of this study, potential variability arising from differences in acquisition protocols and reconstruction settings may influence feature stability, which should be further investigated in future studies.

Before clinical deployment, the model should therefore be evaluated and, if necessary, recalibrated in target populations reflecting local disease prevalence, and the classification threshold should be reassessed for the intended clinical setting.

Third, a direct comparison between the radiomics model and experienced radiologists was not performed. Because this retrospective dataset did not include prospectively recorded, independent, and blinded radiologist assessments for all patients, a reliable head-to-head comparison was not feasible. Therefore, the current findings should not be interpreted as demonstrating superiority of the model over radiologists. A prospective multicenter, multi-reader study comparing radiologists alone, the model alone, and radiologists assisted by the model is required to determine whether the proposed approach provides incremental diagnostic value in clinical practice.

Fourth, the substantially higher training performance compared with the validation and external test cohorts suggests that a certain degree of overfitting may be present. Nevertheless, the decline in external performance is unlikely to be explained solely by overfitting. Calibration analysis in the external test cohort demonstrated acceptable agreement between predicted probabilities and observed outcomes (Brier score = 0.134), supporting the reliability of the model-derived risk estimates. However, the calibration slope below 1 indicated some degree of overconfidence in probability estimation, which may reflect residual model optimism and inter-institutional distributional differences. Importantly, despite these cross-center differences, the model maintained an AUC of 0.803 in the independent external cohort, suggesting that it learned imaging patterns with predictive value beyond the training data rather than merely memorizing cohort-specific characteristics. Nonetheless, the observed reduction in performance indicates that further validation in larger multicenter cohorts and the development of more domain-robust modeling strategies are warranted to improve generalizability. Importantly, although the model was evaluated in an independent external cohort, retrospective validation alone is insufficient to establish its prospective clinical utility. Before routine clinical implementation, the locked model and its classification threshold should be prospectively evaluated in consecutively enrolled patients across multiple centers, scanner platforms, imaging protocols, and clinical settings. Such validation should assess not only discrimination but also calibration, clinical net benefit, subgroup performance, and the incremental value of the model within the real-world diagnostic workflow.

Finally, fp-AML remains uncommon; although this study included a relatively large fp-AML cohort, the sample size limited deeper stratified analyses, for example by tumor size, enhancement pattern, or subtype.

## 5. Conclusions

In summary, we developed and externally validated a triphasic CT-based model with stable cross-center performance. The model may serve as a decision-support tool for the preoperative differentiation of fp-AML from RCC; however, prospective multicenter validation is required before routine clinical implementation.

## Figures and Tables

**Table 1:** Baseline characteristics for patients in discovery and validation sets.

Feature	Training Set N = 364	Internal Test Set N = 156	External Test Set N = 135
Number of CT volumes	1092	468	405
Median age (IQR)	58 (50, 67)	55.5 (48, 64)	56 (48, 63)
Male sex (%)	206 (56.6)	97 (62.2)	86 (63.7)
Surgical approach (%)			
Radical	130 (35.7)	56 (35.9)	40 (29.6)
Partial	229 (62.9)	97 (62.2)	93 (68.9)
Needle biopsy	5 (1.4)	3 (1.9)	2 (1.5)
Pathologic tumor size (%)			
Median (IQR)	3.0 (4.7, 2.0)	3.5 (5.0, 2.2)	3.2 (4.3, 2.1)
≤4	258 (70.9)	99 (63.5)	100 (74.1)
>4 to ≤7	75 (20.6)	37 (23.7)	28 (20.7)
>7 to ;≤10	25 (6.9)	18 (11.5)	7 (5.2)
>10	6 (1.6)	2 (1.3)	0 (0.0)
Imaging appearance (%)			
Cystic	2 (0.5)	1 (0.6)	0 (0.0)
Solid	362 (99.5)	155 (99.4)	135 (100)
TNM stage (%)			
I	246 (84.5)	102 (81.6)	99 (90.8)
II	19 (6.5)	11 (8.8)	5 (4.6)
III	26 (9.0)	12 (9.6)	5 (4.6)
IV	0 (0.0)	0 (0.0)	0 (0.0)
WHO/ISUP Grade ^a^ (%)			
Low (1 & 2)	227 (86.3)	99 (87.6)	73 (73.0)
High (3 & 4)	36 (13.7)	14 (12.4)	27 (27.0)
Necrosis ^a^ (%)	22 (6.0)	14 (9.0)	0 (0.0)
Sarcomatoid differentiation ^a^ (%)	4 (1.1)	1 (0.6)	1 (0.7)
Histologic classification (%)			
Clear cell RCC	252 (69.2)	112 (71.8)	95 (70.4)
Papillary RCC	13 (3.6)	3 (1.9)	5 (3.7)
Chromophobe RCC	19 (5.2)	6 (3.8)	6 (4.4)
Other malignant tumors ^b^	7 (1.9)	4 (2.6)	3 (2.2)
fp-AML	73 (20.1)	31 (19.9)	26 (19.3)

^a^ WHO/ISUP grade was evaluated in clear cell RCC and papillary RCC only. Necrosis and sarcomatoid differentiation were evaluated in malignant tumors. Any sarcomatoid differentiation led to the classification of malignancies. ^b^ Other malignant tumors included collecting carcinoma, clear cell papillary renal cell tumor, multilocular cystic renal neoplasm of low malignant potential, epithelioid angiomyolipoma, TEF-3 rearranged RCC, renal medullary carcinoma, indolent subtypes with sarcomatoid differentiation, mucinous tubular and spindle cell carcinoma, tubulocystic RCC, eosinophilic solid and cystic RCC, etc.

**Table 2:** Diagnostic performance of the model in each cohort.

Cohort	ACC (%)	Sensitivity (%)	Specificity (%)	AUC (95% CI)	F1-Score
Training set	91.4	70.6	100.0	0.868 (0.748–0.940)	0.870
Internal test set	74.4	69.6	93.5	0.868 (0.712–0.948)	0.813
External test set	73.3	72.2	100.0	0.803 (0.741–0.865)	0.900

## Data Availability

The data presented in this study are available upon request from the corresponding author. Restrictions apply due to multi-center patient privacy protection and institutional medical data management regulations of the participating hospitals; raw CT images and clinical case files are archived in the internal server of each affiliated hospital and cannot be publicly released.

## Supplementary Materials

The following supporting information can be downloaded at https://www.mdpi.com/article/10.3390/bioengineering13090967/s1. Figure S1: Diagnostic performance of the model for small renal masses. A. ROC curve for small renal masses in the training set. B. ROC curve for small renal masses in the internal test set. C. ROC curve for small renal masses in the external test set. D. Decision curve analysis of the radiomics model for small renal masses in the external test cohort compared with the treat-all and treat-none strategies. Figure S2: SHAP-based interpretation of the proposed model. A. Global feature importance based on mean absolute SHAP values. B. SHAP summary plot. Figure S3: Calibration performance of the radiomics model in the external cohort. Table S1. Summary of CT Acquisition Parameters Across Different CT Phases and Participating Cohorts. Table S2: LASSO regression selected 25 radiomics features relevant for classification.

## Abbreviations

The following abbreviations are used in this manuscript:

Fp-AML	Fat-Poor Angiomyolipoma
RCC	Renal Cell Carcinoma
CT	Computed Tomography
ROC	Receiver Operating Characteristic
ACC	Accuracy
LASSO	Least Absolute Shrinkage and Selection Operator
DCA	Decision Curve Analysis
