# Supplementary material for: Differentiating Fat-Poor Angiomyolipoma from Renal Cell Carcinoma Using Contrast-Enhanced CT
Source: Bioengineering (Basel). 2026 Aug 24;13(9):967. doi: 10.3390/bioengineering13090967 (PMC13603207; doi:10.3390/bioengineering13090967)
Supplement: Supplementary file 1 [file bioengineering-13-00967-s001.zip › Table S1.pdf]

Table S1. Summary of CT Acquisition Parameters Across Different CT Phases and Participating Cohorts.

CT Phase	Cohort	Manufacturer	Manufactuer Model Name	Reconstructure Kernel	Slice Thickness	Xray Tube Current	N
A Phase	Zhongshan Hospital	GE MEDICAL SYSTEMS	LightSpeed VCT	STANDARD	5-5 (5±0, N=8)	198-250 (214.12±20.02, N=8)	8
		Philips	Ingenuity CT	B	5-5 (5±0, N=2)	208-313 (260.5±74.25, N=2)	2
		SIEMENS	SOMATOM Definition	D30s, B31f	3-5 (4±1.15, N=4)	203-305 (269.33±57.5, N=3)	4
		SIEMENS	SOMATOM Definition AS	B31f	3-5 (4.55±0.84, N=49)	69-620 (290±144.75, N=49)	49
		SIEMENS	SOMATOM Definition AS+	B30f, B31f, B20f	3-5 (4.87±0.5, N=163)	169-659 (411.29±128.05, N=163)	163
		SIEMENS	SOMATOM Definition Flash	B30f	5-5 (5±0, N=1)	116-116 (116±0, N=1)	1
		SIEMENS	SOMATOM Force	Br40d\3	5-5 (5±0, N=1)	150-150 (150±0, N=1)	1
		SIEMENS	SOMATOM go.All	Br40f\3	3-5 (4.33±1.15, N=3)	80-190 (126.33±57.01, N=3)	3
		SIEMENS	Sensation 16	B31f	5-5 (5±0, N=9)	144-244 (208±32.05, N=9)	9
		TOSHIBA	Aquilion ONE	FC18	3-5 (4.41±0.93, N=27)	100-212 (127.78±31.69, N=27)	27
		UIH	uCT 550	B_SOFT_B	3-5 (4.81±0.58, N=47)	82-377 (232.62±65.27, N=47)	47
		UIH	uCT 760	B_SOFT_B	2-5 (4.55±0.91, N=98)	105-537 (287.88±99.36, N=97)	98
		UIH	uCT 780	B_SOFT_B	4-4 (4±0, N=1)	153-153 (153±0, N=1)	1
		UIH	uCT 790	B_SOFT_B	5-5 (5±0, N=1)	198-198 (198±0, N=1)	1
		UIH	uCT 960+	B_SOFT_B	3-5 (4±1.41, N=2)	453-516 (484.5±44.55, N=2)	2
	Xiamen Hospital	GE MEDICAL SYSTEMS	Discovery CT750 HD	STANDARD	1.25-1.25 (1.25±0, N=13)	288-599 (479.69±110.56, N=13)	13
		Philips	iCT 256	B	1-1 (1±0, N=2)	442-628 (535±131.52, N=2)	2
		SIEMENS	SOMATOM Definition Flash	B30f	5-5 (5±0, N=2)	109-195 (152±60.81, N=2)	2
		TOSHIBA	Aquilion ONE	FC18	3-5 (4.5±0.93, N=8)	80-130 (90.88±18.83, N=8)	8
		UIH	uCT 760	B_SOFT_B	3-5 (4.78±0.67, N=9)	166-537 (313.11±160.51, N=9)	9
	Lianyungang People's Hospital	GE MEDICAL SYSTEMS	Discovery CT750 HD	STANDARD	1.25-1.25 (1.25±0, N=20)	208-599 (400.9±127.76, N=20)	20
		GE MEDICAL SYSTEMS	LightSpeed VCT	STANDARD	5-5 (5±0, N=6)	215-280 (269.17±26.54, N=6)	6
		GE MEDICAL SYSTEMS	Optima CT660	STANDARD	10-10 (10±0, N=1)	319-319 (319±0, N=1)	1
		GE MEDICAL SYSTEMS	Optima CT670	STANDARD	5-5 (5±0, N=1)	191-191 (191±0, N=1)	1
		GE MEDICAL SYSTEMS	Revolution CT	STANDARD	0.62-0.62 (0.62±0, N=2)	405-514 (459.5±77.07, N=2)	2
		Philips	iCT 256	B	1-1 (1±0, N=10)	376-706 (491.7±102.13, N=10)	10
	Zhejiang Hospital	GE MEDICAL SYSTEMS	Discovery CT750 HD	STANDARD	1.25-1.25 (1.25±0, N=1)	424-424 (424±0, N=1)	1
		GE MEDICAL SYSTEMS	LightSpeed VCT	STANDARD	5-5 (5±0, N=2)	121-350 (235.5±161.93, N=2)	2
		GE MEDICAL SYSTEMS	Optima CT540	STANDARD	5-5 (5±0, N=1)	230-230 (230±0, N=1)	1

		GE MEDICAL SYSTEMS	Revolution CT	STANDARD	2.5-5 (2.78±0.81, N=18)	249-581 (388.06±111.51, N=18)	18
		GE MEDICAL SYSTEMS	Revolution Frontier	STANDARD	2.5-5 (3.75±1.44, N=4)	399-399 (399±0, N=4)	4
		Philips	Brilliance 64	B	5-5 (5±0, N=1)	356-356 (356±0, N=1)	1
		Philips	iCT 256	B, A	1-5 (2.59±0.92, N=92)	213-908 (594.1±152.03, N=92)	92
		SIEMENS	SOMATOM Definition Flash	B30f	5-5 (5±0, N=3)	132-229 (166.33±54.35, N=3)	3
		SIEMENS	SOMATOM Force	Br40d\3	3-3 (3±0, N=12)	110-473 (208.67±105.49, N=12)	12
		TOSHIBA	Aquilion PRIME	FC13	5-5 (5±0, N=1)	200-200 (200±0, N=1)	1
	First People's Hospital of Lianyungang	GE MEDICAL SYSTEMS	Discovery CT750 HD	STANDARD	1.25-1.25 (1.25±0, N=3)	349-593 (434.67±137.27, N=3)	3
		GE MEDICAL SYSTEMS	LightSpeed VCT	STANDARD	5-5 (5±0, N=1)	250-250 (250±0, N=1)	1
		GE MEDICAL SYSTEMS	Optima CT680 Series	STANDARD	5-5 (5±0, N=19)	114-420 (256.32±91.55, N=19)	19
		Philips	iCT 256	B	1-1 (1±0, N=1)	628-628 (628±0, N=1)	1
		SIEMENS	SOMATOM Definition Flash	I30f\3, B30f	5-5 (5±0, N=6)	240-336 (312.17±40.04, N=6)	6
**V Phase**	Zhongshan Hospital	GE MEDICAL SYSTEMS	LightSpeed VCT	STANDARD	5-5 (5±0, N=8)	199-251 (214.75±19.82, N=8)	8
		Philips	Ingenuity CT	B	5-5 (5±0, N=2)	189-284 (236.5±67.18, N=2)	2
		SIEMENS	SOMATOM Definition	D30s, B31f	3-5 (4±1.15, N=4)	202-305 (269.33±58.35, N=3)	4
		SIEMENS	SOMATOM Definition AS	B31f	3-5 (4.55±0.84, N=49)	69-637 (284.33±148.39, N=49)	49
		SIEMENS	SOMATOM Definition AS+	B30f, B31f	3-5 (4.87±0.5, N=163)	174-659 (398.35±125.17, N=163)	163
		SIEMENS	SOMATOM Definition Flash	B30f	5-5 (5±0, N=1)	113-113 (113±0, N=1)	1
		SIEMENS	SOMATOM Force	Br40f\3	5-5 (5±0, N=1)	150-150 (150±0, N=1)	1
		SIEMENS	SOMATOM go.All	Br40f\3	3-5 (4.33±1.15, N=3)	89-190 (129.33±53.48, N=3)	3
		SIEMENS	Sensation 16	B31f	5-5 (5±0, N=9)	147-252 (205.11±28.04, N=9)	9
		TOSHIBA	Aquilion ONE	FC18	3-5 (4.41±0.93, N=27)	100-212 (135.59±36.55, N=27)	27
		UIH	uCT 550	B_SOFT_B	3-5 (4.81±0.58, N=47)	78-376 (232.53±66.76, N=47)	47
		UIH	uCT 760	B_SOFT_B	2-5 (4.56±0.89, N=98)	95-537 (282.24±98.08, N=97)	98
		UIH	uCT 780	B_SOFT_B	4-4 (4±0, N=1)	149-149 (149±0, N=1)	1
		UIH	uCT 790	B_SOFT_B	5-5 (5±0, N=1)	199-199 (199±0, N=1)	1
		UIH	uCT 960+	B_SOFT_B	3-5 (4±1.41, N=2)	454-490 (472±25.46, N=2)	2
	Xiamen Hospital	GE MEDICAL SYSTEMS	Discovery CT750 HD	STANDARD	1.25-1.25 (1.25±0, N=13)	287-599 (450.85±114.64, N=13)	13
		Philips	iCT 256	B	1-1 (1±0, N=2)	447-652 (549.5±144.96, N=2)	2
		SIEMENS	SOMATOM Definition Flash	B30f	5-5 (5±0, N=2)	109-189 (149±56.57, N=2)	2
		TOSHIBA	Aquilion ONE	FC18	3-5 (4.5±0.93, N=8)	80-130 (90.62±18.98, N=8)	8
		UIH	uCT 760	B_SOFT_B	3-5 (4.78±0.67, N=9)	166-537 (315±159.03, N=9)	9
	Lianyungang People's Hospital	GE MEDICAL SYSTEMS	Discovery CT750 HD	STANDARD	1.25-1.25 (1.25±0, N=20)	187-599 (386.35±114.84, N=20)	20
		GE MEDICAL	LightSpeed VCT	STANDARD	5-5 (5±0, N=6)	215-280 (269.17±26.54, N=6)	6

		SYSTEMS					
		GE MEDICAL SYSTEMS	Optima CT660	STANDARD	10-10 (10±0, N=1)	320-320 (320±0, N=1)	1
		GE MEDICAL SYSTEMS	Optima CT670	STANDARD	5-5 (5±0, N=1)	191-191 (191±0, N=1)	1
		GE MEDICAL SYSTEMS	Revolution CT	STANDARD	0.62-0.62 (0.62±0, N=2)	405-509 (457±73.54, N=2)	2
		Philips	iCT 256	B	1-1 (1±0, N=10)	382-706 (495.6±100.55, N=10)	10
	Zhejiang Hospital	GE MEDICAL SYSTEMS	Discovery CT750 HD	STANDARD	1.25-1.25 (1.25±0, N=1)	409-409 (409±0, N=1)	1
		GE MEDICAL SYSTEMS	LightSpeed VCT	STANDARD	5-5 (5±0, N=2)	116-350 (233±165.46, N=2)	2
		GE MEDICAL SYSTEMS	Optima CT540	STANDARD	5-5 (5±0, N=1)	230-230 (230±0, N=1)	1
		GE MEDICAL SYSTEMS	Revolution CT	STANDARD	1.25-5 (2.71±0.88, N=18)	249-581 (374.72±104.27, N=18)	18
		GE MEDICAL SYSTEMS	Revolution Frontier	STANDARD	2.5-5 (3.75±1.44, N=4)	399-399 (399±0, N=4)	4
		Philips	Brilliance 64	B	5-5 (5±0, N=1)	356-356 (356±0, N=1)	1
		Philips	iCT 256	B, A	1-5 (2.59±0.92, N=92)	217-1003 (688.14±186.53, N=92)	92
		SIEMENS	SOMATOM Definition Flash	B30f	5-5 (5±0, N=3)	130-229 (167.33±53.8, N=3)	3
		SIEMENS	SOMATOM Force	Br40d\3	3-3 (3±0, N=12)	109-493 (210.58±110.25, N=12)	12
		TOSHIBA	Aquilion PRIME	FC13	5-5 (5±0, N=1)	200-200 (200±0, N=1)	1
	First People's Hospital of Lianyungang	GE MEDICAL SYSTEMS	Discovery CT750 HD	STANDARD	1.25-1.25 (1.25±0, N=3)	319-592 (426±145.75, N=3)	3
		GE MEDICAL SYSTEMS	LightSpeed VCT	STANDARD	5-5 (5±0, N=1)	250-250 (250±0, N=1)	1
		GE MEDICAL SYSTEMS	Optima CT680 Series	STANDARD	5-5 (5±0, N=19)	115-419 (254.42±88.76, N=19)	19
		Philips	iCT 256	B	1-1 (1±0, N=1)	633-633 (633±0, N=1)	1
		SIEMENS	SOMATOM Definition Flash	I30f\3, B30f	5-5 (5±0, N=6)	240-336 (312.83±39.61, N=6)	6
**N Phase**	Zhongshan Hospital	GE MEDICAL SYSTEMS	LightSpeed VCT	STANDARD	5-5 (5±0, N=8)	198-222 (204.38±9.02, N=8)	8
		Philips	Ingenuity CT	B	5-5 (5±0, N=2)	187-281 (234±66.47, N=2)	2
		SIEMENS	SOMATOM Definition	B31f	3-5 (4±1.15, N=4)	206-315 (259±59.05, N=4)	4
		SIEMENS	SOMATOM Definition AS	B31f	3-5 (4.55±0.84, N=49)	79-573 (285.35±146.21, N=49)	49
		SIEMENS	SOMATOM Definition AS+	B30f, B31f	3-5 (4.89±0.46, N=163)	168-611 (288.66±78.52, N=163)	163
		SIEMENS	SOMATOM Definition Flash	B30f	5-5 (5±0, N=1)	142-142 (142±0, N=1)	1
		SIEMENS	SOMATOM Force	Br40f\3	5-5 (5±0, N=1)	148-148 (148±0, N=1)	1
		SIEMENS	SOMATOM go.All	Br40f\3	3-5 (4.33±1.15, N=3)	88-190 (129±53.86, N=3)	3
		SIEMENS	Sensation 16	B31f	5-5 (5±0, N=9)	135-245 (204.89±29.33, N=9)	9

	TOSHIBA	Aquilion ONE	FC18	3-5 (4.41±0.93, N=27)	100-212 (127.78±31.69, N=27)	27
	UIH	uCT 550	B_SOFT_B	3-5 (4.81±0.58, N=47)	37-376 (253.64±77.03, N=47)	47
	UIH	uCT 760	B_SOFT_B	2-5 (4.61±0.87, N=98)	100-537 (281.95±100.73, N=97)	98
	UIH	uCT 780	B_SOFT_B	5-5 (5±0, N=1)	155-155 (155±0, N=1)	1
	UIH	uCT 790	B_SOFT_B	5-5 (5±0, N=1)	197-197 (197±0, N=1)	1
	UIH	uCT 960+	B_SOFT_B	3-5 (4±1.41, N=2)	307-375 (341±48.08, N=2)	2
Xiamen Hospital	GE MEDICAL SYSTEMS	Discovery CT750 HD	STANDARD	1.25-5 (1.54±1.04, N=13)	191-549 (340.15±105.33, N=13)	13
	Philips	iCT 256	B	1-1 (1±0, N=2)	445-642 (543.5±139.3, N=2)	2
	SIEMENS	SOMATOM Definition AS+	B30f	5-5 (5±0, N=1)	199-199 (199±0, N=1)	1
	SIEMENS	SOMATOM Definition Flash	B30f	5-5 (5±0, N=1)	133-133 (133±0, N=1)	1
	TOSHIBA	Aquilion ONE	FC18	3-5 (4.5±0.93, N=8)	80-130 (90.88±18.83, N=8)	8
	UIH	uCT 760	B_SOFT_B	3-5 (4.78±0.67, N=9)	166-535 (309.67±158.4, N=9)	9
Lianyungang People's Hospital	GE MEDICAL SYSTEMS	Discovery CT750 HD	STANDARD	1.25-1.25 (1.25±0, N=20)	111-473 (309.65±113.01, N=20)	20
	GE MEDICAL SYSTEMS	LightSpeed VCT	STANDARD	5-5 (5±0, N=6)	215-280 (263.83±27.13, N=6)	6
	GE MEDICAL SYSTEMS	Optima CT660	STANDARD	10-10 (10±0, N=1)	350-350 (350±0, N=1)	1
	GE MEDICAL SYSTEMS	Optima CT670	STANDARD	5-5 (5±0, N=1)	191-191 (191±0, N=1)	1
	GE MEDICAL SYSTEMS	Revolution CT	STANDARD	1.25-1.25 (1.25±0, N=2)	191-524 (357.5±235.47, N=2)	2
	Philips	iCT 256	B	1-1 (1±0, N=10)	404-876 (535.9±141.74, N=10)	10
Zhejiang Hospital	GE MEDICAL SYSTEMS	Discovery CT750 HD	STANDARD	1.25-1.25 (1.25±0, N=1)	270-270 (270±0, N=1)	1
	GE MEDICAL SYSTEMS	LightSpeed VCT	STANDARD	5-5 (5±0, N=2)	202-355 (278.5±108.19, N=2)	2
	GE MEDICAL SYSTEMS	Optima CT540	STANDARD	5-5 (5±0, N=1)	230-230 (230±0, N=1)	1
	GE MEDICAL SYSTEMS	Revolution CT	STANDARD	2.5-5 (2.79±0.83, N=17)	249-546 (359.94±104.6, N=17)	17
	GE MEDICAL SYSTEMS	Revolution Frontier	STANDARD	2.5-5 (3.75±1.44, N=4)	399-399 (399±0, N=4)	4
	Philips	Brilliance 64	B	5-5 (5±0, N=1)	356-356 (356±0, N=1)	1
	Philips	iCT 256	B	2.5-5 (3.77±1.01, N=93)	156-694 (374.85±129.91, N=93)	93
	SIEMENS	SOMATOM Definition Flash	B30f	5-5 (5±0, N=3)	111-272 (186.33±81, N=3)	3
	SIEMENS	SOMATOM Force	Br40d\3	3-3 (3±0, N=12)	141-492 (247±103.18, N=12)	12
	TOSHIBA	Aquilion PRIME	FC13	5-5 (5±0, N=1)	200-200 (200±0, N=1)	1
First People's Hospital	GE MEDICAL SYSTEMS	Discovery CT750 HD	STANDARD	1.25-1.25 (1.25±0, N=3)	204-418 (291.67±112.12, N=3)	3
	GE MEDICAL	LightSpeed VCT	STANDARD	5-5 (5±0, N=1)	250-250 (250±0, N=1)	1

of	SYSTEMS					
Lianyungan	GE MEDICAL	Optima CT680 Series	STANDARD	5-5 (5±0, N=19)	245-374 (261.95±37.19, N=19)	19
g	SYSTEMS					
	Philips	iCT 256	B	1-1 (1±0, N=1)	627-627 (627±0, N=1)	1
	SIEMENS	SOMATOM Definition AS	B30f	5-5 (5±0, N=1)	652-652 (652±0, N=1)	1
	SIEMENS	SOMATOM Definition Flash	B30f	5-5 (5±0, N=5)	240-336 (316.8±42.93, N=5)	5
