# Supplementary material for: Differentiating Fat-Poor Angiomyolipoma from Renal Cell Carcinoma Using Contrast-Enhanced CT
Source: Bioengineering (Basel). 2026 Aug 24;13(9):967. doi: 10.3390/bioengineering13090967 (PMC13603207; doi:10.3390/bioengineering13090967)
Supplement: Supplementary file 1 [file bioengineering-13-00967-s001.zip › Table S2.pdf]

Table S2: LASSO regression selected 25 radiomics features relevant for classification.

No	The Name of RF	The defination of RF
1	A_original_glcm_Idmn	Inverse Difference Moment Normalized of GLCM in the arterial phase
2	A_original_gldm_DependenceEntropy	Dependence Entropy of GLDM in A phase
3	V_original_glcm_Idn	Inverse Difference Normalized GLCM in V phase
4	V_original_glszm_ZonePercentage	ZonePercentage of GLSZM in V phase
5	A_original_ngtdm_Coarseness	Coarseness of NGTDM in A phase
6	V_original_glszm_GrayLevelNonUniformityNormalized	Gray Level Non-Uniformity Normalized GLSZM in V phase
7	N_original_firstorder_RootMeanSquared	Root Mean Squared Intensity in N phase
8	A_original_glcm_Imc1	Informational Measure of Correlation (IMC) 1 of GLCM in A phase
9	A_original_glcm_DifferenceEntropy	Difference Entropy of GLCM in the arterial phase
10	A_original_glrlm_GrayLevelNonUniformityNormalized	Normalized Gray Level Non Uniformity of GLRLM in A phase
11	N_original_glcm_Idn	Normalized Inverse Difference of GLCM in N phase
12	A_original_glszm_LowGrayLevelZoneEmphasis	Low Gray Level Emphasis of GLSZM in A phase
13	N_original_firstorder_Median	Median Intensity in N phase
14	A_original_firstorder_Minimum	Minimum Intensity in A phase
15	V_original_firstorder_Minimum	Minimum Intensity in V phase
16	A_original_firstorder_10Percentile	10th percentile of Intensity in A phase
17	N_original_glrlm_RunLengthNonUniformityNormalized	Run Length Non-Uniformity Normalized GLRLM in N phase
18	N_original_gldm_DependenceEntropy	Dependence Entropy of GLDM in N phase
19	V_original_glszm_SizeZoneNonUniformityNormalized	Size-Zone Non-Uniformity Normalized of GLSZM in the venous phase
20	A_original_glcm_Idn	Inverse Difference Normalized GLCM in A phase
21	V_original_glrlm_RunLengthNonUniformityNormalized	Run Length Non-Uniformity Normalized GLRLM in V phase
22	V_original_glrlm_LongRunEmphasis	Long Run Emphasis of GLRLM in V phase
2	V_original_glcm_Imc1	Informational Measure of Correlation (IMC) 1 of

3		GLCM in V phase
24	V_original_firstorder_Range	The Range of Intensity values in V phase
25	A_original_firstorder_Range	The Range of Intensity values in A phase

- GLDM — Gray-Level Dependence Matrix
- GLRLM — Gray Level Run Length Matrix
- GLCM — Gray Level Co-occurrence Matrix
- NGTDM — Neighbourhood Gray-Tone Difference Matrix
- GLSZM — Gray-Level Size Zone Matrix
